# Feasibility and Accuracy of HIV Testing of Children by Caregivers Using Oral Mucosal Transudate HIV Tests

**DOI:** 10.1097/QAI.0000000000002644

**Published:** 2021-02-01

**Authors:** Chido Dziva Chikwari, Victoria Simms, Katharina Kranzer, Stefanie Dringus, Rudo Chikodzore, Edwin Sibanda, Karen Webb, Nicol Redzo, Hilda Mujuru, Tsitsi Apollo, Getrude Ncube, Karin Hatzold, Sarah Bernays, Helen A. Weiss, Rashida A. Ferrand

**Affiliations:** a Clinical Research Department, London School of Hygiene and Tropical Medicine, London, United Kingdom;; b Biomedical Research and Training Institute, Harare, Zimbabwe;; c MRC Tropical Epidemiology Group, London School of Hygiene and Tropical Medicine, London, United Kingdom;; d Division of Infectious and Tropical Medicine, Medical Centre of the University of Munich, Munich, Germany;; e Ministry of Health and Child Care, Bulawayo, Zimbabwe;; f Health Services Department, Bulawayo, Zimbabwe;; g Organization for Public Health Interventions and Development, Harare, Zimbabwe;; h Department of Paediatrics, University of Zimbabwe, Harare, Zimbabwe;; i AIDS and TB Unit, Ministry of Health and Child Care, Harare, Zimbabwe;; j Population Services International, Harare, Zimbabwe;; k Global Health Department, London School of Hygiene and Tropical Medicine, London, United Kingdom; and; l School of Public Health, University of Sydney, Sydney, Australia.

**Keywords:** HIV, self-testing, children, adolescents, caregiver

## Abstract

Children encounter multiple barriers in accessing facilities. HIV self-testing using oral mucosal transudate (OMT) tests has been shown to be effective in reaching hard-to-reach populations. We evaluated the feasibility and accuracy of caregivers conducting HIV testing using OMTs in children in Zimbabwe.

**Methods:**

We offered OMTs to caregivers (>18 years) living with HIV to test children (2–18 years) living in their households. All caregivers were provided with manufacturer instructions. In Phase 1 (January–December 2018, 9 clinics), caregivers additionally received a demonstration by a provider using a test kit and video. In Phase 2 (January–May 2019, 3 clinics), caregivers did not receive a demonstration. We collected demographic data and assessed caregiver's ability to perform the test and interpret results. Caregiver performance was assessed by direct observation and scored using a predefined checklist. Factors associated with obtaining a full score were analyzed using logistic regression.

**Results:**

Overall 400 caregivers (83.0% female, median age 38 years) who were observed tested 786 children (54.6% female, median age 8 years). For most tests, caregivers correctly collected oral fluid [87.1% without provider demonstrations (n = 629) and 96.8% with demonstrations (n = 157), *P* = 0.002]. The majority correctly used a timer (90.3% without demonstrations and 96.8% with demonstrations, *P* = 0.02). In multivariate logistic regression caregivers who obtained a full score for performance were more likely to have received a demonstration (odds ratio 4.14, 95% confidence interval: 2.01 to 8.50).

**Conclusions:**

Caregiver-provided testing using OMTs is a feasible and accurate HIV testing strategy for children. We recommend operational research to support implementation at scale.

## Abstract 2

Supplemental Digital Content is Available in the Text.

## INTRODUCTION

Globally, 1.8 million children (0–14 years) were estimated to be living with HIV in 2019, but over 845,000 of these were either undiagnosed or diagnosed but not on treatment.^1^ Although coverage of prevention of mother-to-child transmission programs has risen substantially in the last decade, only 50% of exposed infants underwent HIV testing within the first 2 weeks of birth in 2019, and postnatal transmission continues to occur.^2^ Thus, children continue to be infected (150,000 infections in 2019), with many children not presenting to health care until older childhood when they have developed advanced HIV disease.^1,3–5^

Facility-based HIV testing is routinely implemented in most high prevalence settings. However, children encounter multiple unique barriers in accessing facility-based HIV testing services including reliance on caregivers to take them, guardian consent, distance to and costs incurred to access facilities, and inconvenient opening times coinciding with school hours.^6,7^

HIV self-testing (HIVST) using oral mucosal transudate (OMT) tests has been shown to be effective in reaching previously hard-to-reach populations including men, adolescents, sex workers, and men who have sex with men.^8–10^ Benefits of HIVST include privacy and autonomy, decreased workload for health care workers, and improved access through community distribution.^11^

The World Health Organization^12^ (WHO) already recommends community-based HIV testing using OMT tests by lower cadre health care workers, and an extension of this would be HIV testing of children performed by caregivers using OMT. HIV testing for children provided by a caregiver, if feasible and acceptable, could address the barriers to testing children,^13^ thus potentially increasing coverage of HIV testing among children, and decrease the demands on health care provider time and potentially be more cost-effective.^14^

Qualitative studies show that caregivers are willing to perform HIV testing on their children and believe that this form of testing has several advantages including privacy, convenience, control over who knows the child's status, and lower costs.^13,15^ However, some caregivers expressed uncertainty about their ability to test children without assistance and support from a health care worker.^13^ Before recommending HIV testing by caregivers at scale, there is a need to understand whether HIV testing by caregivers is performed correctly.

In this study, we evaluated the feasibility and accuracy of caregivers conducting HIV tests using OMT in children aged 2–18 years in Zimbabwe.

## METHODS

The study was embedded within the Bridging the Gap in HIV Testing and Care for Children in Zimbabwe (B-GAP) project conducted between January 2018–May 2019 in 12 primary care clinics: 9 in Bulawayo (urban) and 3 in Matebeleland South province (rural).^16^ Adult (aged 15–64) HIV prevalence is 18% in Bulawayo and 22% in Matebeleland South.^17^

The B-GAP project aimed to investigate different approaches for index-linked testing for children. Index-linked HIV testing is a strategy whereby an HIV test is offered to contacts of individuals living with HIV. B-GAP evaluated 3 approaches for index-linked HIV testing for children, namely, facility-based testing, home-based testing performed by a lay worker, or testing performed by a caregiver using an OMT test.^18^ This study evaluates testing performed by caregivers.

### Study Participants and Procedures

Study participants were consenting individuals (age ≥ 18 years) living with HIV attending the study clinics for HIV care who had children (2–18 years) in their household of unknown HIV status and who selected the caregiver-provided HIV testing option to test their children. Caregivers had to be biological parents or caregivers (where parents were not available). The unknown HIV status was defined as never having had an HIV test or having a negative test result more than 6 months previously. Caregivers were provided with HIV self-test OMT kits (OraQuick ADVANCE Rapid HIV 1/2, OraSure Technologies Inc.) for each eligible child.^19^ Each kit included manufacturer's instructions in English and the 2 main local languages, Shona and Ndebele. Caregivers were explicitly told that the HIV OMT test is a screening test and that a reactive result would require confirmation by a blood test at the health care facility as per national guidelines.^20^ Caregivers were also provided with a hotline number to contact should they have any questions or concerns during the testing process. All caregivers who took up caregiver testing were followed up (in person or by telephone).

The study was conducted in 2 phases initially as part of the main B-GAP study in 2018 (Phase 1) and as an extension of B-GAP in 2019 to evaluate caregiver testing without demonstrations (Phase 2).

#### Phase 1: Caregivers Received Demonstrations

**FIGURE 1.**
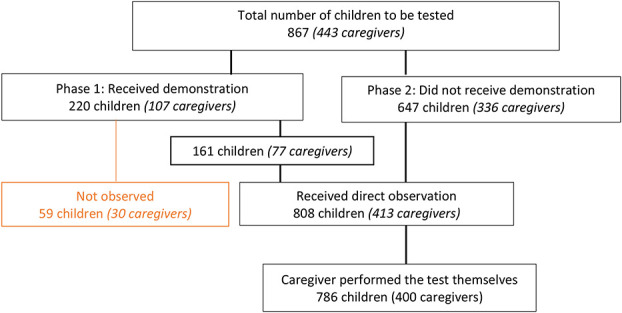
Participant flow chart.

Recruitment in this phase was in 6 urban clinics and 3 rural clinics. From January–December 2018, caregivers who consented to participate in the study and chose to test their children using OMT received a demonstration by research assistants in the clinic of how to perform the HIV self-test (see Fig. 1, Supplemental Digital Content 1, http://links.lww.com/QAI/B607). To demonstrate, the research assistants used the OMT test instruction pamphlets, a dummy test kit, and a 4-minute video on a handheld mobile device. After the demonstration, caregivers were asked to show the research assistant how to perform the test using a dummy kit to check their understanding.

A home visit was scheduled within 5 days for the first 15 caregivers enrolled in each of the 9 facilities. During the home visit, a member of the research team observed the caregiver performing the test (direct observation) on each child. No further demonstrations were provided on the testing day unless the caregiver asked for assistance. If requested to do so, the research assistants could provide assistance to caregivers. All other caregivers were followed up by telephone on day 5 after they had been given the OMT tests to ascertain the test outcome. If not reachable by telephone, caregivers were followed up by home visit (up to 2 home visits).

#### Phase 2: Caregivers Did Not Receive Demonstrations

As most caregivers were able to correctly preform the test in Phase 1; from January to May 2019, caregivers did not receive a demonstration in person or by video in the clinic. Direct observation of the caregiver conducting the test on their child at home was scheduled for all caregivers. During this phase, caregivers were discouraged from asking for assistance from the research assistants but would be assisted if they requested assistance. Recruitment was in 3 urban clinics. No rural clinics were included because of budget constraints.

#### Direct Observation

Direct observation of caregiver testing was performed by research assistants during the scheduled home visits. Using a tablet-based form, research assistants collected data on a predefined checklist assessing the testing process. The checklist evaluated caregiver performance for each step including whether or not the caregiver looked at the instruction pamphlet, collection of oral fluid from the child's mouth, and handling of test kit components such as the buffer and use of a timer when performing the test. Caregivers were asked to interpret the OMT test result. For all directly observed tests, data on whether or not the caregivers interpreted the test result correctly (according to the manufacturer's guidance) were collected. The research assistants also collected data on whether or not the caregivers asked for assistance, what the assistance was for, and when assistance was provided.

After preliminarily data analysis, from March 2019, the research team introduced a more detailed questions asking for the OMT result interpretation by the caregiver and the OMT result interpretation by the research assistant (see Fig. 1, Supplemental Digital Content 1, http://links.lww.com/QAI/B607). In the initial questionnaire, only the caregiver interpretation was captured followed by a question about whether or not this interpretation was correct according to the research assistant. After March 2019, the questionnaire captured caregivers' interpretation of the OMT test result and in addition the interpretation by the research assistant (considered the gold standard). For the entire duration of the study, all children of caregivers who were directly observed when testing the children had a blood-based rapid HIV test performed on the child by the research assistant to confirm the OMT result. All children who tested HIV positive were referred to their preferred health facility for onward care.

Caregiver performance of conducting the HIV test was evaluated using 4 indicators which are part of the steps described in the manufacturer's instructions^19^: (1) Correct collection of oral fluid from the child's mouth ie, gently swab completely around the outer gums, both upper and lower, one time around, using the flat pad; (2) Complete insertion of the flat pad into the buffer solution ie, making sure that the flat pad touches the bottom of the vial; (3) Use of a timer during the test; (4) Correct interpretation of the test result

Each indicator was given a score of 1 if conducted correctly or 0 if incorrect, with a maximum score of 4. In both phases, direct observations and data collection were conducted for all tests conducted in the household that is for each child in the household.

### Data Analysis

Analyses were conducted using STATA v15·0 software (StataCorp, TX). Categorical variables were summarized as counts (percentages), and continuous variables were summarized as medians (interquartile range: IQR). Only tests performed by caregivers who received direct observation were included in the analysis. These caregivers were grouped as those who received a demonstration and those who did not receive a demonstration. We compared 3 key caregiver characteristics (sex, age, and education level) between those who received a demonstration and those who did not, using χ^2^ tests. Similarly, we compared child characteristics (age, sex, and relationship to the caregiver) by whether the caregiver received a demonstration or not, using logistic regression adjusting for clustering by caregiver with robust standard errors.

Univariable and multivariable logistic regression at the child level was used to assess factors associated with obtaining a full score for performance. We adjusted for clustering by caregiver using robust standard errors.

### Ethical Considerations

Ethical approval for this study was obtained from the Medical Research Council of Zimbabwe and the Institutional Review Boards of the Biomedical Research and Training Institute and the London School of Hygiene and Tropical Medicine. Written informed consent was obtained from all caregivers. Verbal assent was obtained from children aged 2–7 years. Written assent was obtained from children aged 7–12 years, and adolescents aged 13–18 years provided signed consent.

## RESULTS

### Demographics and Flow

Between January 2018 and May 2019, 867 children (54.1% female, median age 8 years, IQR 5–12 years) were to be tested by 443 caregivers (81.9% female, median age 38 years, IQR 32–45) (Fig. 1). Of the 443 caregivers, 30 (6.8%) received a demonstration and were not directly observed, 77 (17.4%) received a demonstration and were directly observed, and 336 (75.8%) did not receive a demonstration and were directly observed (Fig. 1). Among the 107 caregivers in Phase 1, 10 (9.3%) were from the rural sites. As planned, all 336 caregivers in Phase 2 were from urban clinics, did not receive demonstrations from the research assistants, and were all directly observed. Overall, 413 caregivers received direct observation, and 400 caregivers (96.9%) performed the test on the children themselves (Fig. 1).

Most of the 400 caregivers who tested their children were female (83.0%), had secondary level education (75.0%), and were the biological parents of the children they tested (70.8%) (Table 1). The median age of these caregivers was 38 years (IQR 32, 45). Receiving a demonstration was associated with caregiver having less education (*P* = 0.06) and caregiver age category (*P* = 0.06) (Table 1).

**TABLE 1.** Demographic Characteristics of Caregivers and Children Who Received Direct Observation and Performed the Test Themselves, Comparing Caregivers Who Received Demonstrations and Those Who Did Not

	Characteristics of Caregivers Who Performed the Test			
	Total, N = 400 (%)	Received Demonstration, N = 74 (%)	Did Not Receive Demonstration, N = 326 (%)	P*
Sex				
Male	68 (17.0)	10 (13.5)	58 (17.9)	0.38
Female	332 (83.0)	64 (86.5)	268 (82.2)	
Age				
18–30	78 (19.5)	10 (13.5)	68 (20.9)	0.06
31–50	267 (66.8)	58 (78.3)	209 (64.1)	
>50	55 (13.8)	6 (8.1)	49 (15.0)	
Education†				
Primary	59 (14.8)	17 (23.3)	42 (12.9)	0.06
Secondary	298 (74.7)	51 (69.9)	247 (75.8)	
Tertiary	42 (10.5)	5 (6.9)	37 (11.4)	

	Characteristics of Children Tested by Caregivers			
	Total, N = 786 (%)	Caregiver Received Demonstration,N = 157 (%)	Caregiver Did Not Receive Demonstration, N = 629 (%)	*P*‡
Sex§				
Male	356 (45.4)	70 (44.9)	286 (45.5)	0.89
Female	428 (54.6)	86 (55.1)	342 (54.5)	
Age§				
2–5	214 (27.3)	38 (24.4)	176 (28.0)	0.17
6–9	244 (31.1)	43 (27.6)	201 (32.0)	
10–15	278 (34.2)	62 (39.7)	206 (32.8)	
16–18	58 (7.4)	13 (8.3)	45 (7.2)	
Relationship to index§				
Biological child	556 (70.8)	99 (63.5)	457 (72.7)	0.12
Nonbiological child	228 (29.0)	57 (36.5)	171 (27.2)	

* *P*-value obtained for χ^2^ tests.

† Education data missing for 1 caregiver.

‡ *P*-value for the child characteristics were obtained from logistic regression adjusting for clustering by a caregiver using robust standard errors.

§ Missing data for 2 children.

Among 808 tests where caregivers were observed, caregivers performed 786 tests themselves (97.3%) (157 who received demonstration and 629 who did not receive demonstrations) (Table 2).

**TABLE 2.** Performance of Caregivers on Each Test Comparing Those Who Received a Demonstration and Those Who Did Not

N = 786	Did Not Receive Demonstration 629 (%)	Received Demonstration 157 (%)	P*
1. Caregiver correctly collected oral fluid	548 (87.1)	152 (96.8)	0.002
2. Caregiver inserted the flat pad all the way	612 (97.3)	156 (99.4)	0.157
3. Caregiver used a timer	568 (90.3)	152 (96.8)	0.019
4. Caregiver correctly interpreted the test result	612 (97.3)	153 (97.5)	0.843
5. Caregiver received a score of 4/4	490 (77.9)	145 (92.4)	<0.001

* *P*-value obtained from logistic regression adjusting for clustering by a caregiver using robust standard errors.

The reasons for not performing the test on the remaining 22 children included the child wanted to perform the test themselves (n = 6), at the point of testing the caregiver said they could not perform the test (n = 7), and that the child wanted the research assistant to perform the test (n = 3). Most of the caregivers who did not perform the test themselves (18/22, 81.8%) had not received a demonstration. Of the 786 children tested, 54.6% were female, and the median age was 8 years (IQR 5, 12). No child characteristics were associated with test demonstration (Table 1).

The subsequent analyses focus on the 786 tests performed by caregivers who were directly observed and performed the test themselves (Fig. 1).

### Performance

For the 786 tests performed by caregivers, caregivers correctly collected oral fluid for most tests, and this was associated with receiving a demonstration (87.1% among those without a demonstration and 96.8% with a demonstration, *P* = 0.002). Among 86 caregivers who did not correctly collect oral fluid, 35 (40.7%) swabbed either the lower or the upper gum alone, scrubbed the gums rather than swabbing, or swabbed both the upper and lower gums more than once. Other caregiver inconsistencies included swabbing front gums alone, brushing teeth rather than swabbing, and placing the flat pad on the tongue or gums. Most caregivers inserted the flat pad all the way into the test fluid (97.3% without a demonstration and 99.4% with a demonstration, *P* = 0.16). Most caregivers used a timer during the test. Caregivers who had received a demonstration were more likely to use a timer (96.8% with and 90.3% without provider demonstrations, *P* = 0.02). Interpretation of the test result was correct in almost all instances (97.3% without a demonstration and 97.5% with a demonstration, *P* = 0.84) (Table 2).

Overall, 635/786 (80.8%) tests were performed correctly on all 4 indicators (490/629–77.9% among those without a demonstration and 145/157–92.4% among those with a demonstration) (Table 2). In univariate analysis adjusting for clustering by a caregiver, having received a demonstration was associated with performing all 4 indicators correctly (Table 3). In multivariable analysis adjusting for clustering by a caregiver, higher level of education when compared with primary level [secondary odds ratio (OR) 2.51; 95% confidence interval (CI): 1.26 to 4.99 and tertiary OR 2.82; 95% CI: 0.91 to 8.79; *P* = 0.06] and receiving a demonstration (OR = 4.14; 95% CI: 2.01 to 8.50; *P* < 0.001) was associated with a full score of 4. Because of their association with having a demonstration, level of education and caregiver age category were included in multivariable analysis. Site (rural vs urban) was not included in this analysis because no rural clinics were included in Phase 2 of the study.

**TABLE 3.** Factors Associated With Obtaining a Full Score for Caregiver-Provided Testing (N = 786)

Characteristic	Obtained a Full Score, n = 635	Univariate Analysis*		Multivariate Analysis†	
	n (%)	OR (95% CI)	*P*	OR (95% CI)	*P*
Index level variables					
Caregiver's age					
18–30	118 (81.9)	1.42 (0.61 to 3.26)	0.63	1.06 (0.45 to 2.53)	0.81
31–50	424 (81.5)	1.37 (0.68 to 2.76)		0.93 (0.44 to 1.97)	
>50	93 (76.2)	1	—	1	—
Caregiver's sex					
Male	101 (80.8)	1	—		
Female	534 (80.8)	1.00 (0.52 to 1.93)	1.00		
Caregiver's highest level of education‡					
Primary	82 (70.7)	1	—	1	—
Secondary	479 (82.3)	1.93 (1.00 to 3.71)	0.12	2.51 (1.26 to 4.99)	0.06
Tertiary	73 (83.9)	2.16 (0.70 to 6.66)		2.82 (0.91 to 8.79)	
Received demonstration					
No	490 (77.9)	1	—	1	—
Yes	145 (92.4)	3.43 (1.79 to 6.58)	<0.001	4.14 (2.01 to 8.50)	<0.001
Child level variables					
Child’ sex§					
Male	288 (80.9)	1	—		
Female	345 (80.6)	0.98 (0.69 to 1.40)	0.92		
Child's age,§ yrs					
2–5	176 (82.2)	1	—		
6–9	192 (78.7)	0.80 (0.51 to 1.23)	0.56		
10–15	223 (83.2)	1.06 (0.66 to 1.74)			
16–18	42 (72.4)	0.56 (0.29 to 1.11)			
Child's relationship to index§					
Nonbiological child	447 (80.4)	1	—		
Biological child	186 (81.6)	0.93 (0.54 to 1.60)	0.78		

* Univariate analysis adjusted for clustering by a caregiver.

† Multivariate analysis adjusting for clustering by a caregiver, caregiver level of education, and caregiver age category.

‡ Missing data for 1 caregiver.

§ Missing data for 2 children.

### Test Results and Caregiver Interpretation

All the caregivers with more detailed interpretation data presented here had not received a demonstration, and interpretation data were available for 587/786 tests. When the 587 OMT tests were compared with blood-based test results, the sensitivity and specificity of the OMT interpretation by research assistants was 100%. All invalid tests (n = 7) were HIV negative using blood-based testing. Of the 567 OMT tests deemed nonreactive by the research assistant (ie, the gold standard), all were identified as nonreactive by the caregivers (specificity = 100%, 95% CI: 99.4% to 100%; Table 4). Of the 13 OMT tests deemed reactive by the caregiver, 4 OMT tests were identified as reactive by the research assistant (sensitivity 30.8%, 95% CI: 9.1% to 61.4%). In addition, one caregiver read a nonreactive test as invalid.

**TABLE 4.** Interpretation of Oral HIV Test Results (N = 587)

	Caregiver Interpretation			Total
	Reactive	Nonreactive	Invalid	
Research assistant interpretation				
Reactive	4	0	0	**4**
Nonreactive	8	567	1	**576**
Invalid	1	0	6	**7**
Total	**13**	**567**	**7**	**587**

Most tests were performed with no assistance from the research assistants, 608/647 (94.0%) among those who did not receive any demonstration and 123/161 (76.4%) among children whose caregiver received demonstrations (*P* < 0.001). Most caregivers who asked for assistance did so when collecting the swab on the child followed by how to maneuver the packaging and test equipment then reading the instructions.

All 30 caregivers who did not have direct observation reported nonreactive results for the 59 children tested.

### Ease of Performing the Test

When caregivers who did not receive a demonstration were asked to score the ease of performing the test from 1 to 5 (1 = very easy to 5 = very difficult), most caregivers reported that performing the test was very easy (41.2%) or easy (34.1%). Only 1.6% of caregivers said that performing the test was difficult. Most caregivers who performed the test used the manufacturer's instructions in vernacular (Shona or Ndebele) (74.9%).

## DISCUSSION

Our study findings show that caregivers can perform oral HIV tests appropriately on their children. Most caregivers in our study were able to accurately collect oral fluid, maneuver test kit components, and correctly interpret test results. We found that prior demonstration of OMT testing by a provider did improve performance, particularly caregiver's ability to correctly swab the child's mouth and also the use of a timer while conducting the test. We also found that most caregivers in our study, who did not receive prior demonstration from providers on how to perform the test, felt that performing the test on their children was easy and usually did not ask for assistance from a health care worker who was present to observe them performing the test. Caregivers were more likely to ask for assistance when they were explicitly told they could ask for assistance. When discouraged from asking for assistance most caregivers were able to perform the test without asking for help. These findings are consistent with other studies where individual's ability to perform the OMT test on themselves has been evaluated in different settings including in South Africa and Zimbabwe, with over 90% of users being able to correctly interpret their HIV test result.^21,22^

Although most caregivers could correctly interpret the oral HIV test result, some caregivers incorrectly interpreted a nonreactive OMT test result as reactive, and there were some invalid test results, likely because of poor performance of the caregivers in conducting the test. No caregivers incorrectly interpreted a reactive OMT test result, although it is important to note that the number of reactive OMT test results was low. Most errors (incorrectly identifying a nonreactive test as reactive) would have been picked up in subsequent confirmatory HIV testing at a health care facility according to WHO HIVST guidelines.^23^ This, however, requires the guardian and child to present to a health care facility for confirmatory testing which may not always be the case. It is also important to consider the emotional impact of believing the test result is reactive on the caregiver and their child. This can be investigated further in operational studies of caregiver-provided HIV testing. We do note, however, that in Phase 1 of our study, we did have caregivers who took OMT kits home to test their children and were not observed as would happen in routine implementation.

OMT testing for HIV is accurate in children and is now recommended by WHO and international implementing partners, such as PEPFAR, as a screening test for individuals aged 2 years and above.^14,24^ Caregiver-provided HIV testing, such as self-testing, if rolled out is likely to be performed independently at home without any providers present. In a previous qualitative work from our group caregivers have expressed fears about making a mistake while performing the test and concerns about not having a health care provider present to support them; however, all package inserts from the OMT manufacturer do contain local hotline numbers for remote assistance.^13^ This study shows that the most caregivers can perform the test independently without support from providers and also without prior demonstrations.

In our formative work, caregivers were also worried about dealing with negative reactions to an HIV positive result.^13^ As with HIVST in adults, concerns about social harms in the form of intimate partner violence or gender-based violence are warranted. In HIVST studies among adults' social harm reports were infrequent in Malawi.^9,25^ Our study had a very low HIV prevalence, however, among the children tested and those who were diagnosed with HIV no social harms were reported.

Key limitations of our study design are that demonstration was not allocated at random, and hence, the 2 groups that received and did not receive test demonstration may not have been comparable. This was due to the sampling strategy whereby the study was conducted in 2 phases, and an urban population was oversampled for the group that did not receive demonstrations. Another limitation of our study is possible observer bias. Caregivers may have performed the test better or more confidently because they knew they were observed. We also note the low number of HIV-positive children in our study as a limitation precluding any firm conclusion of interpretation of reactive results. Although our study does provide the first evidence for this testing strategy, we recommend further evaluations of caregiver-provided testing to facilitate collection of good surveillance and operational data to assess when, if, how, and where to roll out large scale caregiver-provided testing.

This study shows that task shifting from highly skilled health workers such as nurses in health facilities to caregiver may be a feasible strategy for earlier diagnosis of HIV in children.^12^ This is a timely approach in the context of the ongoing COVID-19 pandemic where lockdowns have made it more difficult to access health facilities for any service including HIV testing.^26^ In addition to collection from health facilities, OMT kits can be distributed in the community as was piloted in another study in Malawi.^27^ Another key consideration for caregiver-provided testing is rapid and effective home linkage to HIV care for children who test HIV positive. Concerns about linkage to care in the context of adult HIVST programs have been raised; however, linkage to care can be supported in multiple ways including telephone or community follow-up and provision of incentives.^27,28^ Evidence is limited, and further studies to assess linkage to and retention in care for children diagnosed through caregiver provider testing are warranted.

Acceptability of caregiver-provided testing must also be assessed. This testing strategy may not be acceptable for all caregivers because of its novelty which may require sensitization efforts before scale up. In addition, caregiver-provided testing may not be suitable for testing older adolescents who may not want to disclose their own sexual activity to caregivers.^18^ Further evaluations of which user groups to target such as female caregivers reiving antenatal care in facilities, individuals already receiving care but with untested children, or individuals newly diagnosed with HIV as is performed with sexual partner testing may be useful.

The cost-effectiveness of caregiver-provided testing should also be assessed and should include assessments of the potential cost savings through reduced skilled health care worker time in facilities. At scale, health worker time may involve only distribution of OMT tests to caregivers from facilities and time to demonstrate OMT test use as was performed in Phase 1 of our study. In our study, lay workers with 3 weeks training on HIV testing and HIV self-testing through the Ministry of Health and Child Care in Zimbabwe conducted the demonstration thus indicating feasibility for this to be performed by lower cadre health workers.

We conclude that caregiver-provided testing is a feasible HIV testing strategy for children and recommend further operational research to support implementation at scale.
